# Genome wide survey, discovery and evolution of repetitive elements in three Entamoeba species

**DOI:** 10.1186/1471-2164-9-595

**Published:** 2008-12-10

**Authors:** Hernan Lorenzi, Mathangi Thiagarajan, Brian Haas, Jennifer Wortman, Neil Hall, Elisabet Caler

**Affiliations:** 1 J. Craig Venter Institute, 9704 Medical Center Drive, Rockville, MD, 20850, USA; 2 Broad Institute, 7 Cambridge Center, Cambridge, MA, 02142, USA; 3 University of Maryland School of Medicine, 655 West Baltimore Street, Baltimore, MD, 21201, USA; 4 School of Biological Sciences, University of Liverpool, Liverpool, L69 7ZB, UK

## Abstract

**Background:**

Identification and mapping of repetitive elements is a key step for accurate gene prediction and overall structural annotation of genomes. During the assembly and annotation of three highly repetitive amoeba genomes, *Entamoeba histolytica*, *Entamoeba dispar*, and *Entamoeba invadens*, we performed comparative sequence analysis to identify and map all class I and class II transposable elements in their sequences.

**Results:**

Here, we report the identification of two novel *Entamoeba*-specific repeats: ERE1 and ERE2; ERE1 is spread across the three genomes and associated with different repeats in a species-specific manner, while ERE2 is unique to *E. histolytica*. We also report the identification of two novel subfamilies of LINE and SINE retrotransposons in *E. dispar *and provide evidence for how the different LINE and SINE subfamilies evolved in these species. Additionally, we found a putative transposase-coding gene in *E. histolytica *and *E. dispar *related to the *mariner *transposon *Hydargos *from *E. invadens*. The distribution of transposable elements in these genomes is markedly skewed with a tendency of forming clusters. More than 70% of the three genomes have a repeat density below their corresponding average value indicating that transposable elements are not evenly distributed. We show that repeats and repeat-clusters are found at syntenic break points between *E. histolytica *and *E. dispar *and hence, could work as recombination hot spots promoting genome rearrangements.

**Conclusion:**

The mapping of all transposable elements found in these parasites shows that repeat coverage is up to three times higher than previously reported. LINE, ERE1 and *mariner *elements were present in the common ancestor to the three *Entamoeba *species while ERE2 was likely acquired by *E. histolytica *after its separation from *E. dispar*. We demonstrate that *E. histolytica *and *E. dispar *share their entire repertoire of LINE and SINE retrotransposons and that Eh_SINE3/Ed_SINE1 originated as a chimeric SINE from Eh/Ed_SINE2 and Eh_SINE1/Ed_SINE3. Our work shows that transposable elements are organized in clusters, frequently found at syntenic break points providing insights into their contribution to chromosome instability and therefore, to genomic variation and speciation in these parasites.

## Background

*Entamoeba *species comprise a group of unicellular eukaryotes that include parasitic organisms that infect humans. In particular, *E. histolytica *is the etiological agent responsible for amoebic dysentery and liver abscess leading to the death of hundreds of thousands of people annually. *E. dispar*, a closely related non-pathogenic species, is morphologically identical to *E. histolytica *but with very different pathogenic properties [1]. Both species are able to colonize humans but only *E. histolytica *is able to cause invasive disease. Behavior such as tissue damage and erythrophagocytosis is not seen with *E. dispar in vivo*. *E. invadens *is a reptilian parasite used as a model of encystation for *E. histolytica*, as *E. invadens *will form cysts in axenic cultures. *E. invadens *affects several reptile taxons, causing disease in squamates (scaled reptilians), and also causing significant morbidity and mortality in chelonians (turtles).

Recently, new efforts have been made to improve the current *E. histolytica *genome assembly and annotation, and to complete the genome sequences for *E. dispar *and *E. invadens*. For this purpose, and in order to generate accurate gene predictions and annotation, a detail identification of repeat elements in the genome is fundamental. Several families of transposable elements (TEs) have been described for the nuclear genome of these parasites [2-5]. It is well established that transposable elements play an important role in nuclear architecture, genome stability, gene amplification, and altered gene regulation [6-8]. In addition, as mentioned above, identification of repeat elements is essential for correct gene set generation, since unidentified TEs can affect the quality of gene annotation and annotation-dependent analyses such as microarray-based gene expression studies [9]. For this reason, our initial goal was to identify and map all the TEs that populate these three *Entamoeba *genomes.

TEs are conventionally classified into two broad classes, I and II. Class I includes two distinct types of TEs, long terminal repeats (LTR) and non-LTR retroelements, both requiring reverse transcription from an RNA intermediate. LTR retroelements include retroviruses and *Ty1/Ty3*-like retrotransposons, and are reverse transcribed from RNA intermediates, duplicated, and then transposed as double-stranded DNA. Non-LTR retroelements consist of short or long interspersed nuclear elements, respectively SINEs or LINEs [10,11], and are transposed by reverse transcription of mRNA directly into the site of integration. On the other hand, class II TEs comprise elements that transpose DNA (transposons).

Even though TEs can represent a large fraction of the nuclear genome of multicellular organisms [6,8], it is only recently that we have a wealth of finished genome information from unicellular eukaryotes to expand the knowledge about the presence of TEs in protozoan parasites such as *Entamoeba spp*. Recent reports have identified three different subfamilies of LINE and SINE elements and a *mutator*-related DNA transposon in *E. histolytica*; a single family of LINEs, SINEs, and *mutator *transposons in *E. dispar; *and a single LINE family and four types of class II TEs related to transposon superfamilies *Ty1/mariner*, *mutator*, *piggyBac*, and *hAT *in *E. invadens *[2-5].

The reassembly and reannotation of the *E. histolytica *nuclear genome and the completion (5×) of the *E. dispar *and *E. invadens *genome sequences provided us with the necessary framework to perform a survey of the currently known *Entamoeba *transposable elements, and gave us the opportunity to discover previously uncharacterized novel repetitive sequences. We have mapped all known class I and class II transposons in these genomes, revealing that the collection of shared TEs and the abundance of repetitive sequences are much larger than what was previously reported [2]. In addition, we report the identification and characterization of two novel TEs, one of which is specific for *E. histolytica*. The present study provides insights into the evolution and diversity of transposable elements in these parasites and the role they could have played during genomic variation and speciation.

## Methods

### Repeat sequences accessions

Examples of the transposable element sequences from *E. histolytica*, *E. dispar *and *E. invadens *described here have been submitted to GenBank and can be searched under the following accession numbers: Ed_LINE2, EU099436; Ed_SINE2, EU099437; Ed_LINE3, EU099438; Ed_ERE1, EU099439; Ed_SINE3, EU099440; Ed_Hydargos (Ed_mariner in this paper), EU099441; Eh_ERE1, EU099442; Eh_Hydargos (Eh_mariner in this paper), EU099443; EhERE2, EU099444; Ei_ERE1, EU099445; Ei_LINE, EU099446. Consensus sequences for Eh_ERE1, Ed_ERE1, Ei_ERE1, Eh_ERE2, Ed_LINE2 and Ed_SINE2 elements were also submitted to RepBase together with two representative sequences for Ed_SINE3 and Ed_LINE3.

### Genome sequence (data mining)

The genome sequencing for the three *Entamoeba *species were performed in collaboration between the J. Craig Venter Institute, JCVI (formerly The Institute for Genomic Research (TIGR)) and the Sanger Institute. The draft genome for *E. histolytica *was initially published by Loftus et al [12], and was re-assembled and re-annotated at JCVI (manuscript in preparation). The genomes of *E. dispar *and *E. invadens *have been assembled and annotated at JCVI. The new assemblies for *E. histolytica *(AAFB00000000), *E. dispar *(AANV00000000) and *E. invadens *(AANW00000000) have been deposited in GenBank and can be searched through the National Center for Biotechnology Information (NCBI) web site .

### Repeat finders analysis

Several elements have been already characterized in these organisms (Table 1). To survey the genomes, we created a comprehensive custom database containing all reported *Entamoeba *elements from GenBank and Repbase [13] and ran RepeatMasker  to map and quantify the elements. To identify novel repeats we followed two different strategies. The first was to generate a library of highly repetitive sequences for each genome using RepeatScout [14]. Output sequences encoding known proteins or PFAM domains were filtered out. The remaining repeats were surveyed for class I and class II transposable elements with TransposonPSI , a program that performs PSI-BLAST searches using a set of position specific scoring matrices generated from different collections of TE families. The second strategy was to perform all-versus-all genome sequence alignments using nucmer  followed by sequence clustering based on similarity. Clusters containing most repetitive sequences were subsequently selected for further analysis.

**Table 1:** Distribution of transposable elements in *E. histolytica*, *E. dispar *and *E. invadens*

**Element type**	**Genome distribution**		
	*E. histolytica*	*E. dispar*	*E. invadens*

Class I			
LINE	+ (Eh_LINE1^a^, Eh_LINE2^a^, Eh_LINE3^a^)	+ (Ed_LINE1^a^, Ed_LINE2, Ed_LINE3)	+ (Ei_LINE^a^)
SINE	+ (Eh_SINE1^a^, Eh_SINE2^a^, Eh_SINE3^a^)	+ (Ed_SINE1^a^, Ed_SINE2, Ed_SINE3)	-

Class II			
mutator	+ (EMULE^a^)	+ (EMULE^a^)	+ (EMULE^a^, phantom^a^)
hAT	-	-	+ (Chapka^a^)
mariner/Tc1	+ (Hydargos)	+ (Hydargos)	+ (Hydargos^a^, Gemini^a^, Piglet^a^, Mogwai^a^, Gizmo^a^)
piggyBac	-	-	+ (leapFrog^a^)

Unknown	+ (Eh_ERE1, EhERE2)	+ (Ed_ERE1)	+ (Ei_ERE1)

(+), present; (-), absent. Repeat subfamilies are denoted within parenthesis.

^a ^TEs identified in previous reports.

Finally, consensus DNA sequences for each repetitive element identified with either methodology were built from multiple sequence alignments with ClustalX [15]. Extensive manual examination of repeat structures and insertion sites was required for novel elements. Putative ORFs identified with ORF finder  were conceptually translated.

### Non-synonymous/synonymous substitution rate (Dn/Ds ratio)

For a group of repeats containing conserved but non-coding sequences, it is expected no selection at the codon level. To verify the coding capacity of ORFs identified in ERE1 and ERE2 we computed the ratio of non-synonymous to synonymous substitutions (ω) [16,17] on the DNA sequence containing the ORF of interest. For proteins under purifying selection it is expected a ω << 1 while for proteins under strong positive selection the value should be ω >> 1. A ω value close to 1 is an indication that sequences are under no selective pressure and therefore are unlikely to encode proteins [18,19]. To calculate ω, sequences were first aligned using MUSCLE [18] and the program codeml from PAML [19,20] was run using model M0 to calculate the overall (i.e. branch- and position-independent) ω value for the ORF of interest.

### Phylogenetic analysis

Multiple sequence alignments for the different TE families were constructed using ClustalX followed by manual curation when necessary. Next, phylogenetic trees were built by the Neighbor-Joining method using a bootstrap value of 1000. Finally, trees were plotted with TreeView .

### Genome coverage, repeat density and distribution

Genome coverage and copy number estimations for each element were calculated by alignment of each repeat to the assembled genomes using RepeatMasker. Partial contiguous RepeatMasker hits derived from the same TE were counted as a single repeat if they were ordered with respect to the element and mapped less than 100 bp apart from each other. Repeat coverage was estimated with respect to the total length of the respective genomes. Distribution of repeats was analyzed using an in-house Perl script to estimate the frequency and composition of different repeat clusters along the genomes. Repeat densities were estimated per scaffold using the following equation:

$$\delta_{\text{repeats}}=\frac{n\times 10000}{length}$$

where δ represents repeat density expressed as number of repeats every 10 Kb, *n *is the number of repeats per scaffold and *length *is the scaffold length in base pairs.

### Identity- and dot-plots

Construction of identity plots for syntenic regions between *E. histolytica *and *E. dispar *were generated using the PipMaker web resource [21]. Dot-plots analyses were performed with *dotter *[22] using a cutoff value of 60 identical nucleotides every 100 bp.

## Results

### Identification of Entamoeba Repetitive Element 1 (ERE1), a novel repetitive element of *Entamoeba sp*

**Figure 1:**
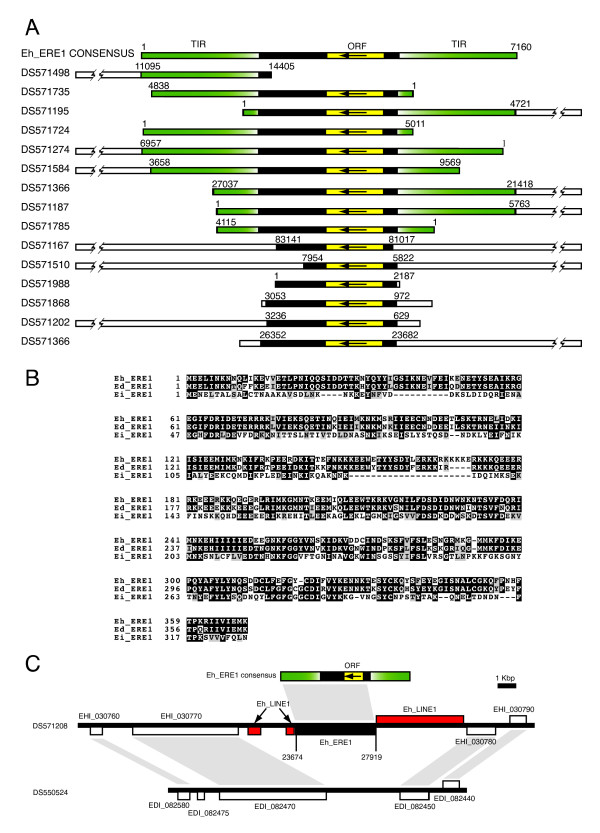
**Identification and characterization of ERE1 in *Entamoeba sp***. A) Reconstruction of Eh_ERE1 consensus sequence from multiple fragmented copies scattered along the *E. histolytica *assembly. Green boxes, flanking Eh_ERE1 terminal inverted repeats (TIR); black boxes, Eh_ERE1 core region; yellow boxes, single Eh_ERE1 ORF where the arrow indicates sense of transcription; white boxes, *E. histolytica *scaffolds. Numbers represent coordinates within scaffolds. GenBank accession numbers of scaffolds are indicated on the left. B) Multiple alignment of the consensus protein sequences coded by Eh_ERE1, Ed_ERE1 and Ei_ERE1. Black-shaded letters, identical residues; gray-shaded letters, conservative changes. C) Syntenic regions from *E. histolytica *(top) and *E. dispar *(bottom) showing an example of Eh_ERE1 transposition. White boxes, protein coding genes; black box, Eh_ERE1; red boxes, LINEs; gray areas, regions of similarity. GenBank locus tags are indicated above or below genes. Scaffold GenBank accessions are shown on the left. Features on the forward or reverse strand are displayed above or below the scaffolds, respectively.

To identify all the repetitive elements present in the *E. histolytica *genome, 1499 scaffolds from the current genomic sequence data were surveyed using RepeatScout or an all-versus-all comparison approach as described in Methods. In a preliminary screening, all known elements and new repetitive sequences above 400 bp were selected for further analysis. Among this collection of repetitive sequences we identified several truncated copies of an AT-rich (82%) repeat that was named ERE1 (Eh_ERE1). To reconstruct the structure of an intact Eh_ERE1 we performed multiple sequence alignments of fragmented copies of the element using ClustalX. The resulting consensus sequence showed that Eh_ERE1 is a 7,160 bp TE composed of an inner 2,719 bp core region flanked by two 2,221 bp terminal inverted repeats (TIR) (Fig. 1A). The core region contained a single open reading frame (ORF) potentially coding for a 369 amino acids protein with an average percent of identity of 72% at the protein level among all Eh_ERE1 TEs. No homologue to this protein was found in the GenBank non-redundant protein database. However, sequence comparisons to the NCBI clusters of orthologous groups of proteins , a database that clusters proteins based on their phylogenetic relationship, showed a weak but consistent similarity (e-value < 9 × 10^-7^) to proteins belonging to two different groups of genes associated to DNA metabolism: chromosome segregation ATPases (COG1196) and ATPases involved in DNA repair (COG419).

Genome distribution analysis showed that Eh_ERE1 is primarily inserted in AT-rich intergenic regions frequently nearby other repetitive elements (see below and Fig. 8B); no instances were found where its integration disrupted a protein-coding gene. Estimation of Eh_ERE1 copy number with RepeatMasker showed that there are 777 fragmented copies of Eh_ERE1 scattered along the *E. histolytica *genome spanning a total of 1 Mb bp of genomic sequence. This estimation includes 100 putative complete elements where truncations coincide with the end of scaffolds (Table 2).

**Table 2:** Number and coverage of transposable elements in *E. histolytica*, *E. dispar *and *E. invadens*

**name**	**complete**^a^	**incomplete**^a^	**coverage (bp)**	**coverage (%)**^b^
Eh_LINE1	88	654	1079630	5.2%
Eh_LINE2	73	442	759971	3.7%
Eh_LINE3	10	87	160940	0.8%
Eh_SINE1	264	181	187972	0.9%
Eh_SINE2	94	162	108195	0.5%
Eh_SINE3	9	40	18851	0.1%
Eh_ERE1	0	777	1014754	4.9%
Eh_ERE2	71	728	733987	3.5%
Eh_MuDR	0	4	2851	< 0.1%
Eh_mariner	0	1	1008	< 0.1%
**Eh_TOTAL**	**609**	**3047**	**4068159**	**19.7%**

Ed_LINE1	63	510	839108	3.7%
Ed_LINE2	28	449	506244	2.2%
Ed_LINE3	2	42	46734	0.2%
Ed_SINE1	282	143	208941	0.9%
Ed_SINE2	53	136	73091	0.3%
Ed_SINE3	2	16	2497	< 0.1%
Ed_ERE1	51	536	526451	2.3%
Ed_MuDR	0	4	2075	< 0.1%
Ed_mariner	0	1	1011	< 0.1%
**Ed_TOTAL**	**481**	**1837**	**2206152**	**9.7%**

Ei_LINE	2	67	59,308	0.1%
Ei_ERE1	30	227	170,510	0.4%
Ei_DDE	328	2607	1,678,976	4.1%
Ei_mariner	390	1400	822,878	2.0%
Ei_hAT	35	755	464,161	1.1%
Ei_MuDR	49	831	522,116	1.3%
Ei_Polinton	5	126	336,005	0.8%
Ei_piggyBac	14	32	27,894	0.1%
**Ei_TOTAL**	**677**	**6082**	**4033163**	**9.9%**

^a ^Repeats were considered complete when their length was at least 90% of the consensus sequence.

^b ^Expressed as percentage of the corresponding genome length.

BLAST searches against the genomes of *E. dispar *and *E. invadens *revealed that ERE1 is also present in these two species, although the overall structural organization of the elements differs from that in *E. histolytica*. In *E. dispar*, ERE1 (Ed_ERE1) is a 3,216 bp element composed of a central 2075 bp region containing a putative ORF and flanked by two inverted Ed_SINE1 sequences. The ORF encodes a 366 aa protein 81% identical at the amino acid level to the one from the consensus Eh_ERE1 (Fig. 1B). It is interesting to note that although in *E. histolytica *Eh_ERE1 is flanked by a different type of TIR, the single copy of Ed_SINE1 identified so far in *E. histolytica *(Eh_SINE3) [2,5] is inserted next to a truncated Eh_ERE1 element that carries the whole coding sequence of the element, resembling the organization of Ed_ERE1 in *E. dispar *(see below and Fig. 8C). Both, the central region of Ed_ERE1 and Ed_SINE1, were found inserted alone throughout the *E. dispar *genome, suggesting that at some point during evolution, these two elements were able to transpose independently from each other. Because the previous observation implies that these two elements might be independent, Ed_ERE1 coverage and copy number were estimated independently from Ed_SINE1. We identified 587 copies of Ed_ERE1 including 51 complete elements, spanning a total of 526 Kb of genomic sequence (Table 2).

Similar analysis in *E. invadens *showed that ERE1 (Ei_ERE1) contains a single putative ORF coding for a 327 aa protein with 30% identity (48% similarity, e-value = 2 × 10^-31^) to the putative Eh_ERE1 peptide (Fig. 1B), but we could not find any large TIR flanking the coding region. Due to the low degree of conservation among Ei_ERE1 TEs, it was not possible to accurately define the boundaries of the element. For this reason, genomic distribution was estimated using the most conserved portion of Ei_ERE1 represented by a 1,759 bp DNA fragment spanning a single ORF. This approach revealed that the genome of *E. invadens *contains 257 copies of Ei_ERE1, 30 of which are complete elements (as defined above), spanning 170 Kb (0.4%) of genomic sequence (Table 2).

To discard that the single gene found in ERE1 is not a spurious ORF caused by the AT-rich composition of this element we computed the ratio of non-synonymous to synonymous substitutions (Dn/Ds) for this gene in each species. If the ORFs were not functional or spurious it would be expected to find a similar number of synonymous and non-synonymous substitutions and therefore, the Dn/Ds ratio (ω) should be close to 1. In the three cases the omega value was significantly lower than 1 (ω_Eh_ERE1 _= 0.49, ω_Ed_ERE1 _= 0.49, ω_Ei_ERE1 _= 0.47), indicating that the gene coded by ERE1 is under purifying selection.

Comparative sequence analysis between these genomes revealed several syntenic regions between *E. histolytica *and *E. dispar *where ERE1 was inserted in one genome but not the other (Fig. 1C) supporting the hypothesis that at some point in the evolution of these species ERE1 was able to transpose, although it is not clear whether it is still active.

### Identification of Entamoeba Repetitive Element 2 (ERE2), a transposon-like element specific to *E. histolytica*

**Figure 2:**
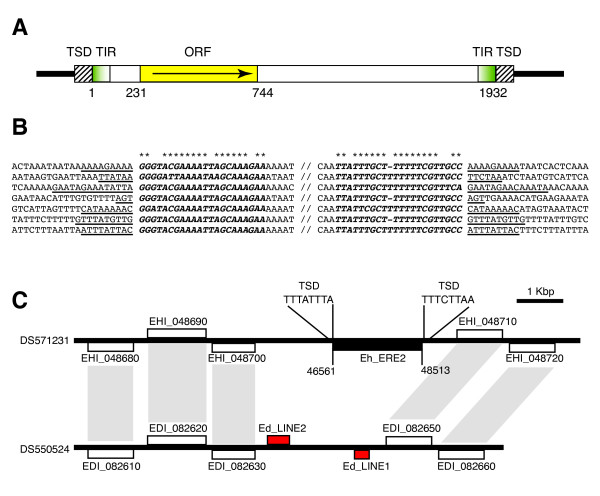
**Characterization of ERE2 in *Entamoeba histolytica***. A) Schematic representation of ERE2. Yellow box, ERE2 open reading frame; white box, ERE2 core region; green boxes, imperfect terminal inverted repeats (TIR); stripped boxes, target site duplications (TSD). B) Multiple sequence alignment of the ERE2 5' and 3' imperfect inverted repeats and insertion sites. Bold letters represent inverted repeats; asterisks denote nucleotides conserved in both TIRs; target site duplications are shown underlined. C) Example of Eh_ERE2 transposition in a syntenic region from *E. histolytica *(top) and *E. dispar *(bottom). White boxes, protein coding genes; black box, Eh_ERE2; red boxes, LINEs. Orthologous pairs of genes are denoted by gray shading. Scaffold GenBank accession numbers are indicated on the left.

Further analysis of the *E. histolytica *genome led us to the identification of a second 1,936 bp repetitive element named ERE2 (Fig 2). Its consensus sequence consists of a 1,892 bp sequence flanked by two 22 bp imperfect TIRs that are 82% identical (Fig. 2A and 2B). The internal fragment contains a putative ORF coding for a 173 aa polypeptide with no homology to any known protein. However, estimation of the Dn/Ds ratio indicated that this ORFs is under purifying selection (ω_Eh_ERE2 _= 0.49) strongly suggesting that it is a real protein coding gene. Interspecies comparative sequence analysis exposed many copies of Eh_ERE2 that are absent in the corresponding syntenic regions from *E. dispar *supporting the hypothesis that Eh_ERE2 is a transposable element (Fig. 2C). HoHA closer examination of the ERE2 integration site indicated that the element inserts preferentially into AT-rich intergenic regions (80% AT). During transposition ERE2 generates 3–14 bp target site duplications (TSD, Fig. 2B), but it does not seem to recognize any specific targeting genomic sequence. We quantified approximately 800 copies of ERE2 in the *E. histolytica *genome, including 71 complete elements (at least 90% of the consensus length), representing a total genomic coverage of 734 Kb (3.5% of the *E. histolytica *genome, Table 2). Contrary to Eh_ERE1, we could not find any copy of ERE2 in the genomes of either *E. dispar *or *E. invadens*, suggesting that *E. histolytica *acquired this TE independently after diverging from *E. dispar*, its closest relative.

### Identification of *mariner *transposons in the genomes of *E. histolytica *and *E. dispar*

**Figure 3:**
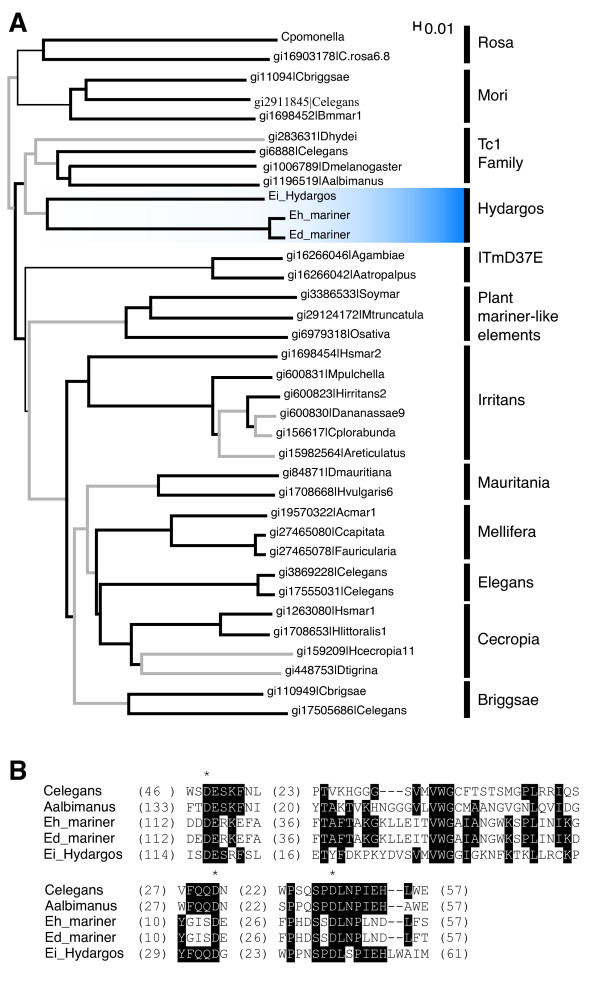
**Identification of mariner-related elements in *E. histolytica *and *E. dispar***. A) Phylogenetic position of Eh_mariner, Ed_mariner and Ei_Hydargos (highlighted in blue) in the *IS630/Tc1/mariner *superfamily. *Mariner *subfamilies and related transposons (Tc1, ItmD37E, and plant *mariner*-like elements) are shown. Elements are identified by host name and GeneInfo Identifier (gi). Branches supported by less than 500 bootstrap replicates are depicted as thin black lines; branches having bootstrap values between 500 and 750 are shown as bold grey lines; branches with values above 750 are represented as bold black lines. B) ClustalX alignment of the transposase domain found in Eh_mariner and Ed_mariner together with three closely related transposases. Amino acids conserved in at least 3 sequences are colored in black. Asterisks denote the three conserved glutamic residues typical of this type of transposases. Parentheses indicate number of residues between conserved blocks.

Pritham et. al [3] reported the existence of five different families of transposons belonging to the *Tc1/mariner *superfamily in the genomes of *E. invadens *and *E. moshkovskii *(Table 1), suggesting that these TEs were already present in the common ancestor of the three *Entamoeba *species in this study. However, no such transposons have been identified in *E. histolytica *and *E. dispar*, raising the question whether *E. invadens *and *E. moshkovskii *acquired these *mariner *transposons by horizontal transfer or vertically from the common ancestor. To address this issue we used Transposon-PSI, an analysis tool developed in-house to identify sequences homologous to large and diverse families of transposable elements (see Methods) to look for *mariner*-related sequences in the genomes of *E. histolytica *and *E. dispar*. The program identified two genomic regions, one from each organism, that gave a highly significant hit (e-value < 1 × 10^-14^) against a *mariner *transposase from *Drosophila melanogaster *(gi1006789 in Fig. 3A). Both regions contained an ORF coding for a 335 aa and a 336 aa protein in *E. histolytica *(Eh_mariner) and *E. dispar *(Ed_mariner) respectively. These putative proteins shared 95% identity throughout their entire sequence suggesting that they could correspond to the same *locus *in both genomes. Further comparative analyses of a 20 Kb genomic region encompassing these ORFs confirmed that Eh_mariner and Ed_mariner were syntenic (data not shown). Unfortunately, it was not possible to determine the precise boundary of the elements due to the short nature of the mariner TIRs (less than 50 bp) [3].

BLASTP searches revealed that Eh_mariner and Ed_mariner were closely related to *Hydargos *(e-value < 4 × 10^-15^), an *E. invadens *transposon that belongs to the *Tc1/mariner *superfamily [3]. This was further confirmed by phylogenetic analysis using the Neighbor-Joining method (bootstrap value = 961/1000, Fig. 3A). As expected, Eh_mariner and Ed_mariner coding proteins contained a D,D33D motif, typical of this type of transposase (Fig. 3B).

### Evolutionary study of LINE elements in *E. histolytica*, *E. dispar *and *E. invadens*

Previous studies have demonstrated the existence of several families of non-LTR retrotransposons related to the R2 group of LINE elements in the genomes of *E. histolytica *(Eh_LINE1, Eh_LINE2, Eh_LINE3)[2,4], *E. dispar *(Ed_LINE1)[4,5] and *E. invadens *(Ei_LINE)[3]. However, to our knowledge, there is no current analysis of how these *Entamoeba *TEs are related to each other. To better understand the evolutionary history of these retrotransposons we mapped all the LINE elements in the three genomes and studied their relationship based on phylogenetic analysis (see Methods). This approach led to the identification of two additional LINE subfamilies in the genome of *E. dispar*. The first element, named Ed_LINE2, shares 86% nucleotide similarity with Eh_LINE2 of *E. histolytica*. Ed_LINE2 integrates into AT-rich intergenic regions generating TSDs at both ends of the element. The consensus Ed_LINE2 is a 4,735 bp TE that contains a putative 5' ORF coding for a 480 aa protein of unknown function and a second ORF that encodes a 956 aa reverse transcriptase protein with a C-terminal domain containing a nucleic acid binding site and a nuclease motif [2]. All the elements identified to date contain multiple stop codons interrupting at least one of the two ORFs suggesting there are no functional copies of Ed_LINE2 in *E. dispar*. The second element, Ed_LINE3, is a 4,406 bp sequence that shares more than 70% identity at the nucleotide level with Eh_LINE3. Contrary to Ed_LINE2, Ed_LINE3 termini are poorly conserved and therefore no flanking TSD could be identified. These results suggest that LINE2 and LINE3 subfamilies were already present in the common ancestor to *E. histolytica *and *E. dispar*.

**Figure 4:**
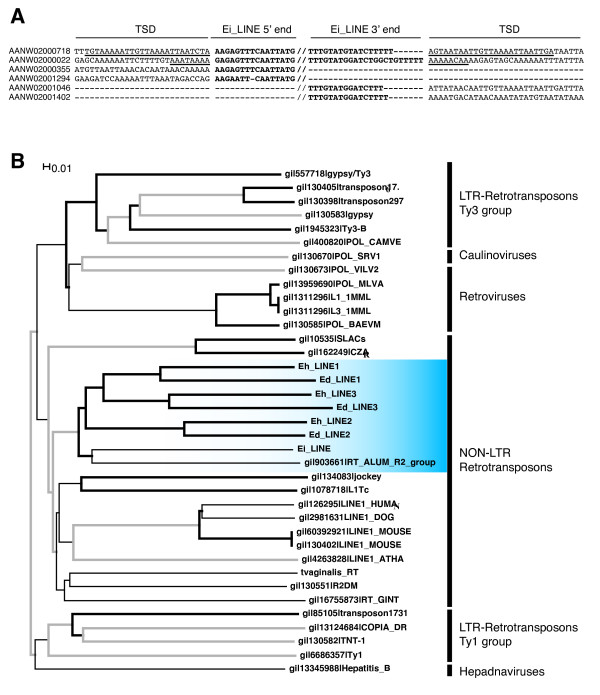
**Characterization and phylogenetic analysis of LINE elements in *Entamoeba sp***. A) Multiple sequence alignment of the 5' and 3' ends of Ei_LINE and insertion sites. The 5' and 3' termini are highlighted in bold. Target site duplications (TSD) are underlined. Ei_LINEs from contigs AANW02000355, AANW02001294, AANW02001046 and AANW02001402 are truncated and lack either the 5' or 3' end of the element. Genomic coordinates for Ei_LINEs excluding ISD are: AANW02000718 (41,801–46,844), AANW02000022 (38,839–43,869), AANW02000355 (5,491–6,819), AANW02001294 (486–1,665), AANW02001046 (2,758-1,949) and AANW02001402 (3,418-2,447). GenBank accessions of *E. invadens *contigs are indicated on the left. B) Phylogenetic analysis of the reverse transcriptase sequences from all identified *Entamoeba *LINEs compared to reverse transcriptases derived from different families of retroelements and retroviruses. Thin black lines, branches with bootstrap values below 500; bold grey lines, branches containing bootstrap values between 500 and 750; bold black lines, branches supported by bootstrap values above 750. Nodes containing *Entamoeba *LINEs are highlighted in blue.

Characterization of the single LINE element previously identified in *E. invadens *[3] indicated that Ei_LINE is a 5,043 bp sequence flanked by TSDs (Fig. 4A). Only two complete copies of Ei_LINE were found in *E. invadens *and neither of them had a complete ORF coding for a reverse transcriptase protein.

Phylogenetic analysis based on manual reconstruction of the reverse transcriptase consensus sequence indicated that all LINEs found in the three *Entamoeba *species were derived from a single ancestral sequence that was already present before they diverged from each other (Fig. 4B). At a later point in evolution, in the ancestral line that led to *E. histolytica *and *E. dispar*, LINE TEs split into two separate lineages giving rise to the Eh/Ed_LINE1 and Eh/Ed_LINE2 subfamilies. Subsequently, in this same ancestor, a third LINE subfamily diverged from LINE1 giving origin to Eh_LINE3 in *E. histolytica *and Ed_LINE3 in *E. dispar *(Fig. 4B).

### Identification of an *E. histolytica *Eh_SINE2-like element in the genome of *E. dispar*

The genomes of *E. histolytica *and *E. dispar *share most of their LINE and SINE families as indicated above [2,3,5]. Indeed, *E. histolytica *LINE1-3 have their counterparts in *E. dispar*: Ed_LINE1[2], and Ed_LINE2- and Ed_LINE3-like sequences (this report); while *E. dispar *Ed_SINE1 is equivalent to *E. histolytica *Eh_SINE3 and closely related to Eh_SINE1. However, to date there is no evidence pointing to the existence of an element equivalent to Eh_SINE2 in *E. dispar*. To verify this observation, we performed BLASTN searches of the *E. dispar *genome with Eh_SINE2 identifying a 604 bp element (Ed_SINE2) 68% identical at the nucleotide level to Eh_SINE2. A multiple sequence alignment of these two elements showed a high degree of conservation at the 5' and 3' ends while the central regions were more divergent. We mapped a total of 189 copies of Ed_SINE2 in *E. dispar *including 53 complete elements (Table 2). These results indicate that all the non-autonomous SINE elements together with the three LINE subfamilies already existed in the common ancestor before the speciation process that gave rise to *E. histolytica *and *E. dispar*.

### Origin of Ed_SINE1/Eh_SINE3 in the genomes of *E. dispar *and *E. histolytica*

**Figure 5:**
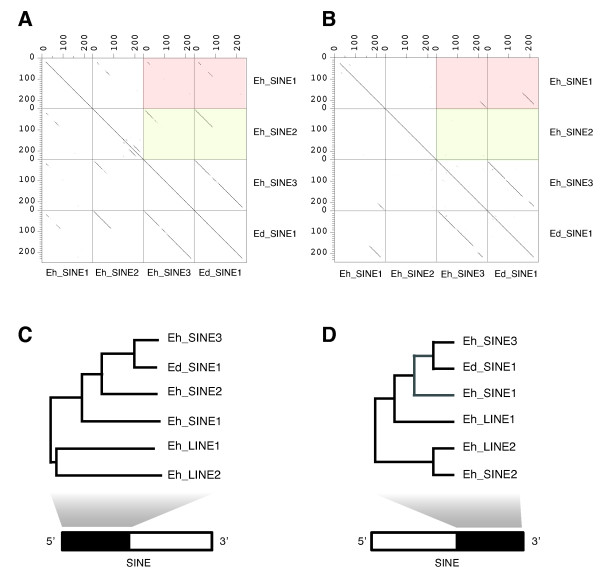
**Evolutionary analysis of Eh_SINE3/Ed_SINE1 in *E. histolytica *and *E. dispar***. A) and B) All-vs-all dot-plot analyses of the first (A) or last (B) 240 bp of Eh_SINE1, Eh_SINE2, Eh_SINE3 and Ed_SINE1. Each dot represents at least 60 identical nucleotides between sequences using a sliding window 100 bp wide. Numbers above or at the left of each dot-plot represent nucleotide positions for each sequence. Comparisons between Eh_SINE1 and either Eh_SINE3 or Ed_SINE1 are highlighted in red, while plots between Eh_SINE2 and either Eh_SINE3 and Ed_SINE1 are highlighted in green. C) and D) Phylogenetic trees showing the relationships between the first 240 bp (B) or last 240 bp (C) of Eh_SINE3/Ed_SINE1 and Eh_SINE1, Eh_SINE2, Eh_LINE1 and Eh_LINE2. Branches supported by bootstrap values between 500 and 750 or above 750 are depicted in grey or black, respectively.

**Figure 6:**
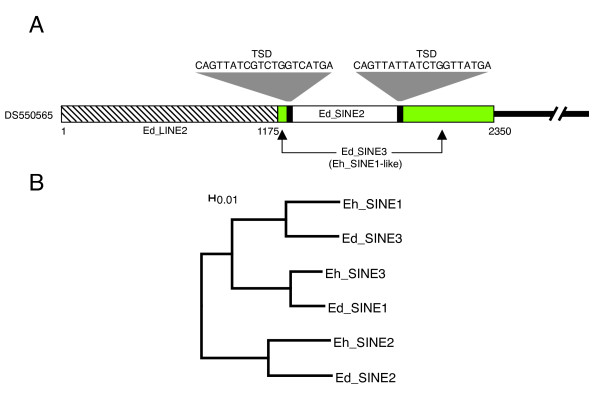
**Phylogenetic analysis of SINE elements in *E. histolytica *and *E. dispar***. A) Schematic representation of an *E. dispar locus *containing a copy of Ed_SINE3 (green boxes) interrupted by the insertion of an Ed_SINE2 (white box) generating target site duplications (TSD, black boxes). The diagonal stripped box represents an Ed_LINE2 located at the end of the scaffold. Scaffold GenBank accession is indicated on the left. B) Phylogenetic analysis of the three SINE families found in *E. histolytica *and *E. dispar*. All tree nodes have a bootstrap value of 1000 (1000 replicates).

Shire et al [5] identified Ed_SINE1 in the genome of *E. dispar*, a SINE element homologous to the single copy of Eh_SINE3 found in *E. histolytica *[2,5]. Nevertheless, the origin of Ed_SINE1/Eh_SINE3 is not clear. To elucidate this issue, we performed dot-plot alignments of Ed_SINE1/Eh_SINE3 against Eh_SINE1 and Eh_SINE2. This analysis showed that the 5' end of Ed_SINE1/Eh_SINE3 is more similar to Eh_SINE2 whereas it's 3' end resembles Eh_SINE1 (Fig. 5A and 5B). This result was subsequently confirmed by phylogenetic analysis using either the first (Fig. 5C) or last (Fig. 5D) 240 bp of these elements together with similar fragments from Eh_LINE1 and Eh_LINE2. This study suggests that in the common ancestor of *E. histolytica *and *E. dispar*, Ed_SINE1/Eh_SINE3 originated as a chimeric element; its 5' end derived from the precursor sequence of Eh_SINE2/Ed_SINE2 (bootstrap = 992/1000) and its 3' end originated from an ancestral Eh_SINE1-like element (bootstrap = 910/1000) (Fig 5C and 5D). If this is the case, then there should be some evidence in *E. dispar *indicating that such ancestral Eh_SINE1-like elements existed in the common ancestor of these two parasites. A survey of the *E. dispar *genome with the sequence from Eh_SINE1 revealed the existence of a 579 bp element with more than 83% identity to Eh_SINE1 (Fig. 6A). This element was interrupted by the insertion of an Ed_SINE2 77 bp from its 5' end generating 21 bp duplications of part of the Eh_SINE1-like sequence at either side of the integration site. Manual reconstruction of the original element, followed by phylogenetic analysis, confirmed that this sequence was closely related to Eh_SINE1 (bootstrap value = 1000/1000; Fig. 6B) and was named Ed_SINE3. The fact that we found only two full-length copies of Ed_SINE3 in *E. dispar *suggests that this element is no longer functional and the remaining copies are just "fossil" sequences derived from a previously active element in the *E. histolytica *and *E. dispar *ancestral genome.

Our repeat survey identified several additional copies of Eh_SINE3 scattered along the *E. histolytica *genome (Table 2). Comparative sequence analysis of regions containing Eh_SINE3 elements in *E. histolytica *showed many cases where their syntenic counterparts in *E. dispar *lacked an Ed_SINE1 inserted in an equivalent position, demonstrating that Eh_SINE3 was able to transpose in *E. histolytica *after its separation from *E. dispar*. Moreover, we found one case where two Eh_SINE3 elements were 99% identical suggesting that the element could be still active.

Altogether, the analysis described above suggests that the pair Ed_SINE1/Eh_SINE3 originated as a chimeric element in the common ancestor of *E. histolytica *and *E. dispar *and was subsequently amplified in *E. histolytica *and, more successfully, in *E. dispar *associated with Ed_ERE1 and Ed_LINE1 or as an independent repeat.

### Representation of transposable elements in *Entamoeba sp*

In a recent comparative analysis of the distribution of LINE and SINE elements in *E. histolytica*, Bakre et al. [2] reported that TEs account for just 6% of this genome. However, that estimation was based on a previous draft of the *E. histolytica *assembly and did not include other TEs identified in this paper. Similarly, Pritham et al [3] reported a quantification of TEs for *E. histolytica*, *E. dispar*, and *E. invadens *based solely on BLAST hits to unassembled reads from the Sanger database. To estimate the abundance of TEs in the current *E. histolytica*, *E. dispar *and *E. invadens *genome assemblies we map all TEs identified to date in these three genomes using RepeatMasker. This analysis revealed that TEs span 4.07 Mb (19.7%) of the *E. histolytica *genome with LINE and SINE retrotransposons representing a total of 11.2% (Table 2). Eh_LINE1 constitutes the largest family of TEs in *E. histolytica *with a total of 742 elements, including 88 complete copies and 46 putative complete elements, truncated due to their location at the end of assemblies. Similarly, Ed_LINE1 is the most frequent LINE in *E. dispar *having 573 copies with 63 complete and 64 putative complete elements that map at the end of assemblies (Table 2).

Consistent with previous reports [2,3], we identified a lower representation of LINE and SINE elements in the genomes of *E. dispar *(1.7 Mb) and *E. invadens *(59.3 Kb). All the LINEs identified in the three genomes have one or more in-frame stop codons or frame-shifts interrupting at least one of their putative protein coding genes. However, since sequence coverage for *E. dispar *and *E. invadens *is 5X, we cannot rule out that some of these mutations are artifacts caused by sequencing errors.

A similar cross-species distribution was found for ERE1, which is more abundant in *E. histolytica *(1 Mb, 777 copies) followed by *E. dispar *(0.5 Mb, 587 copies) and *E. invadens *(0.1 Mb, 257 copies; Table 2). On the other hand, estimation of transposon coverage showed the opposite situation. Indeed, DNA transposons spanned a total of 3.8 Mb of the *E. invadens *genome, while just a few copies of class II elements were found in *E. histolytica *and *E. dispar *(Table 2). This distributional bias observed across the three *Entamoeba *species analyzed in this study confirmed previous quantification results performed on unassembled genomic sequences [3].

### Distribution of transposable elements in *Entamoeba sp*

**Figure 7:**
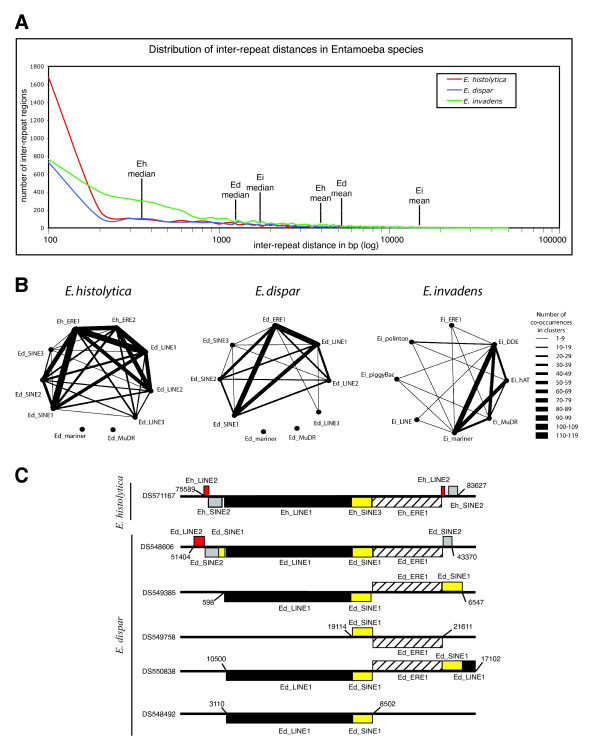
**Distribution analysis of transposable elements in *Entamoeba sp***. A) Distribution of inter-repeat distances in *E. histolytica *(Eh, red), *E. dispar *(Ed, blue) and *E. invadens *(Ei, green). Mean and median values for each species are indicated by vertical lines. Distances were grouped using 100 bp bins. B) Diagram representing the number of simultaneous occurrences of all possible pairs of different repeats within repeat-clusters for each species. Line thickness connecting two different repeats is proportional to the number of times a repeat pair is part of a cluster C) Schematic diagram showing the association among Eh_SINE3/Ed_SINE1, LINE1 and ERE1 in *E. histolytica *and *E. dispar*. This three-component unit composed by LINE1, Ed_SINE1 and ERE1 is found amplified several times in *E. dispar*. GenBank accessions are indicated on the left of the figure. Numbers denote scaffold coordinates. Red boxes, Eh/Ed_LINE2; grey boxes, Eh/Ed_SINE2; yellow boxes, Eh_SINE3/Ed_SINE1; black boxes, Eh/Ed_LINE1; stripped boxes, Eh/Ed_ERE1.

To investigate the distribution of TEs in the three species we plotted the frequency of all inter-repeat distances for each genome using 100 bp bins (Fig. 7A). This analysis showed that inter-repeat distances follow a right-skewed distribution with a median value significantly lower than the mean distance indicating that, in the three Entamoebas, TEs tend to be clustered instead of evenly distributed along the genomes. *E. histolytica *has the most compactly clustered repeats with 50% of the TEs separated by less than 390 bp while median distance values for *E. dispar *and *E. invadens *are 1136 bp and 1634 bp, respectively (Fig. 7A). To determine the composition of repeat-clusters we quantified the number of times all possible pairs of repeats belonged to the same repeat-cluster, defining as repeat-cluster a series of repeats separated by up to 250 bp (Fig. 7B). This quantification showed that in *E. histolytica *Eh_ERE1 is the element most frequently found in the clusters (467 copies) followed by Eh_LINE1 (363 copies) and Eh_ERE2 (336 copies). Most repeat-repeat co-occurrences exist among long TEs with the exception of Eh_ERE1 that also clusters with Eh_SINE1 and Eh_SINE2 at high frequency. There is an evident linkage between Ed_ERE1, Ed_LINE1 and Ed_SINE1 in *E. dipar*, while in *E. invadens *transposons from the DDE and *mariner *families show the maximum number of interactions with other repeats (Fig. 7B).

Further investigation of the numerous interactions between Ed_ERE1, Ed_LINE1 and Ed_SINE1 showed that in *E. dispar *the genomic region syntenic to the *E. histolytica locus *containing the single reported copy of Eh_SINE3 (assemblies DS571167 and DS548606 in Fig. 7C)[2,5] displays a similar repeat organization containing an Ed_SINE1 in-between an Ed_LINE1 and an Ed_ERE1 elements (Fig. 7C). This three-component structure is found amplified 26 times in the genome of *E. dispar *and seen even more frequently is a two-component unit composed of an Ed_SINE1 linked to either Ed_ERE1 or Ed_LINE1 (43 and 22 copies respectively).

**Figure 8:**
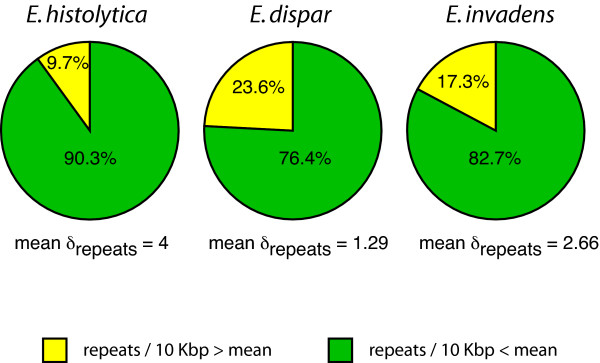
**Repeat densities in *Entamoeba sp***. Proportion of genomic regions with repeat densities below (green) or above (yellow) the average density value for each genome. Repeat densities are expressed as number of repeats every 10 Kb. Scaffolds were positioned into one of the two categories based on their repeat coverage.

An analysis of TE dispersion along the scaffolds revealed that 90% of the genomic sequence in *E. histolytica *has a repeat density of less than 4 repeats every 10 Kb, the mean value for this genome (Fig. 8). A similar uneven repeat density was observed for *E. dispar *and *E. invadens*, where 75% or more of the genomic sequence has a TE content below the average (Fig. 8). These results demonstrate that repeats and repeat-clusters are not uniformly distributed along these genomes but they are enriched in relatively small genomic regions, while most of the three genomes have a TE content below their average density.

## Discussion

In this work we report the first complete whole genome survey of transposable elements for the newly generated assemblies of *E. histolytica*, *E. dispar*, and *E. invadens*. This analysis led to the identification of two novel *Entamoeba*-specific repetitive elements, ERE1 and ERE2, two LINE and two SINE subfamilies in *E. dispar*, and a *mariner*-related sequence in the genomes of *E. histolytica *and *E. dispar*. We also found that TE representation in *E. histolytica *is much higher than previously thought (19.7% in this study versus 6% in [2]). A comparison between repeat coverage in the old and new *E. histolytica *assemblies revealed that both GenBank releases have similar repeat contents (17.4% in the old assembly versus 19.7% in the reassembled genomic sequence). Therefore, the higher repeat coverage reported herein is likely due to two main factors: the use of a more sensitive algorithm to identify repetitive regions (RepeatMasker versus BLAST in [2]) and the discovery of two novel TE families (ERE1 and ERE2) based on intra- and inter-species sequence comparison.

Our analysis also confirms previous results showing that the population of TEs in *E. histolytica *and *E. dispar *is enriched in non-LTR retroelements while *E. invadens *contains mostly class II transposons [2,3,5]. Despite this bias, LINEs, ERE1s, and transposons from *mariner *and *mutator *superfamilies were found in the three genomes indicating they were already present in the common ancestor rather than acquired horizontally after the three species diverged from each other.

Among all the TEs reported in this work, ERE1 and ERE2 are the most intriguing. Both elements have features in common such as a high AT content, the presence of a single ORF, two TIRs delimiting their termini (except for Ei_ERE1), and their integration into intergenic regions (Fig. 1 and 2). Although their putative encoded proteins have no significant hits against any known transposase, their structure resembles that of DNA transposons. In particular, ERE2 is flanked by two 19 bp imperfect TIRs and generates TSDs similar to other known DNA transposons such as Mutyl [23], Pat [24] and Gulliver [25]. The ω values calculated for the ORFs found in ERE1 and ERE2 suggest that both genes are under purifying selection and might still be functional. Moreover, we have found *E. histolytica *EST evidence that suggests that both Eh_ERE1 and Eh_ERE2 are transcribed in this parasite. In addition, comparative sequence analysis between the genomes of *E. histolytica *and *E. dispar *shows many cases where both TEs are found inserted in genomic *loci *from one *Entamoeba *genome but not in their syntenic counterparts from the other species, strongly supporting the idea that these two elements were able to transpose at some point in evolution. Due to its absence in other *Entamoeba *species studied in this report, Eh_ERE2 seems to be the only TE in *E. histolytica *that has been acquired by horizontal transfer.

Given the high degree of conservation between the genomes of *E. histolytica *and its closely related non-pathogenic species *E. dispar*, it is important to ask how *E. histolytica *causes disease. It is well established in prokaryotic organisms that DNA repeats can mediate genomic rearrangements that can alter the expression of disease-associated genes [26]. For example, in pathogenic bacteria such as *Neisseria gonorrhoeae *and *Neisseria meningitidis*, repeat-mediated rearrangements promote positioning of cell surface genes next to "on switches", causing otherwise silent genes to be expressed [27]. Therefore, it is tempting to speculate that the acquisition and posterior amplification of elements such as Eh_ERE2 in the genome of *E. histolytica *has played a role in speciation and acquisition of pathogenicity traits promoting genome rearrangements or altering the function or number of genes involved in processes such as cell attachment, evasion of the host immune response, etc. For example, it has been described that the leucine-rich/BspA-like gene family, that codes for proteins that seem to localize to the parasite plasma membrane and might interact with the host fibronectin [28], is frequently associated to TEs [2]. We have determined that 41 out of 114 of these genes are at less than 1 Kb of a repetitive sequence (data not shown). Currently, we are carrying out studies looking for additional gene families associated to TEs in *E. histolytica*.

Contrary to Eh_ERE2, ERE1 is present in the genomes of the three *Entamoeba *species analyzed in this study and its single ORF encodes a putative protein similar to members from two NCBI COGs composed of proteins associated with DNA repair and recombination. Considering both the structural features of this repetitive element and the fact that the ERE1-encoded polypeptide is similar to proteins involved in DNA metabolism, our data suggest that ERE1 is likely a class II TE. Interestingly, the coding region of ERE1 is associated with different types of terminal repeats depending on the organism. While in *E. histolytica *the ERE1 coding region is generally flanked by the same 2,221 bp TIR, in *E. dispar *ERE1 is often associated to other TEs, mainly Ed_SINE1, and to a lesser extent Ed_LINE1 and Ed_SINE2. Therefore, it is not clear whether Ed_ERE1 is able to transpose independently or if it requires the presence of a nearby repeat. Moreover, 47% of the Eh_ERE1 elements found in *E. histolytica *map within repeat-clusters linked to other TEs (Fig. 7B) and hence, it is possible that the significant expansion of repeats in this parasite had also contributed to the observed 34% increase in the number of Eh_ERE1 repeats compared to the frequency of Ed_ERE1 in the *E. dispar *genome. Lastly, in *E. invadens *Ei_ERE1 is poorly conserved and we were unable to identify any copy associated to other repeats. Both the low copy number and degree of conservation among Ei_ERE1s suggest that this element is not longer functional in *E. invadens*.

Our study also revealed that Eh_SINE3/Ed_SINE1 is a chimeric element where the 5' end was derived from Eh_SINE2/Ed_SINE2 and the 3' end from Eh_SINE1/Ed_SINE3. In addition to the single Eh_SINE3 previously reported for *E. histolytica *[2,5], we could identify at least 9 intact and 40 truncated copies of the element in this genome (Table 2). The identification of Eh_SINE3 elements integrated into *E. histolytica loci *without the presence of a Ed_SINE1 in the corresponding syntenic regions from *E. dispar *(data not shown) indicates that Eh_SINE3 remained active in *E. histolytica *after its separation from *E. dispar*.

Our analysis of the distribution of LINE and SINE retrotransposons in the three *Entamoeba *genomes demonstrates that all LINE elements derived from a common ancestral TE related to the R2 group of LINEs (Fig. 4B, bootstrap value = 926/1000). LINE elements remained silent in the lineage that led to *E. invadens*, but were very active in the ancestor of *E. histolytica *and *E. dispar*, where gave rise to three different LINE and SINE subfamilies. It is not clear whether these non-LTR retroelements are currently active.

Although we identified no LINEs having an intact ORF1 and ORF2, there were some LINE1 and LINE2 elements in *E. histolytica *and *E. dispar *with either a complete ORF1 or ORF2 coding for a putative protein with a reverse transcriptase domain. It has been reported that LINE elements with a non-functional mutation of its reverse transcriptase gene are still able to transpose using the proteins coded by a functional LINE element [29,30]. Therefore, it is likely that these elements are still able to transpose using the required replication machinery in *trans*. Mapping of assembled ESTs onto the *E. histolytica *genome using PASA (Program to Assemble Spliced Alignments) [31], revealed that some copies of Eh_SINE1 and Eh_SINE2 are actually transcribed, but we found no evidence of expressed LINE genes. However, BLAST searches against unassembled *E. histolytica *ESTs identified several ESTs that are at least 98% identical to Eh_LINE1, Eh_LINE2 or Eh_LINE3 supporting the idea that LINEs might still be active in *E. histolytica*.

A striking difference between *E. invadens *and the genomes of *E. histolytica *and *E. dispar *is the compositional bias in their respective TE populations. Our results confirmed previous studies [3] showing that in *E. invadens *most of the collection of repetitive elements is composed of class II transposons while the other two species are rich in class I repeats. Since *E. moshkovskii *and *E. invadens *have a similar set of DNA transposons, Pritham et al [3] proposed that most of these repeats should have been present in the common ancestor and not incorporated horizontally. Thus, there should be some evidence for the previous existence of such elements in the genomes of *E. histolytica *and *E. dispar*. The results presented herein indicate that at least a *mariner *family, related to the transposon *Hydargos *from *E. invadens*, was present in the ancestral *Entamoeba *genome that gave origin to the three species studied in this work. The fact that *E. histolytica *and *E. dispar *only have a single identifiable copy of a *Hydargos*-related element and the low degree of conservation of the residues surrounding the three catalytic aspartic acids of its putative transposase (Fig. 3B) suggests that this TE is not active.

The repeat survey of *E. histolytica*, *E. dispar*, and *E. invadens *reveals a much higher representation of repetitive elements than previously reported [3]. Although it is not clear whether TEs are playing a role in amoeba fitness, it seems likely that the high coverage of repetitive elements in *E. histolytica *(20%) could play a part in the high frequency of genomic variation among the parasite population. This could explain, in part, why less than 25% of the *E. histolytica *infections cause disease [32-35]. Moreover, this study demonstrates that in these genomes transposable elements have a clustered distribution with more than 75% of the genome containing a repeat density below the average, and a small portion of the genome (less than 24%) containing a high repeat density. Repeats and repeat-clusters might work as hot spots for recombination promoting rearrangement between chromosomes and contributing to genome plasticity. Mukherjee et al. [36] have shown that *E. histolytica *is able to change its DNA content and size when switching between xenic and axenic conditions. Although these changes seem to be mainly caused by variations in the number of copies of the entire genome, the authors propose that some specific regions could be differentially amplified or deleted [36]. If that is the case, then it seems plausible that repeats and repeat-clusters may be playing a role in these processes.

**Figure 9:**
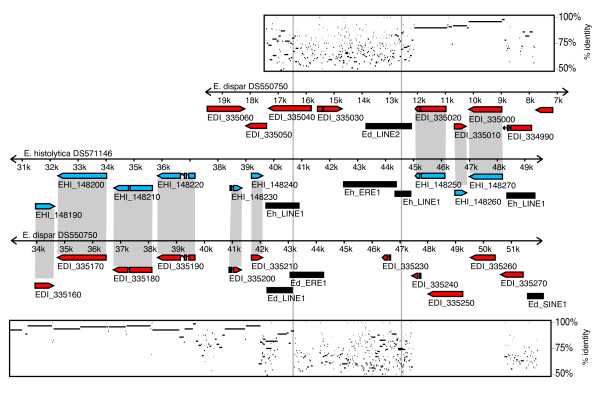
**Example of repeat clusters at a syntenic break between *E. histolytica *and *E. dispar***. Black boxes, repetitive elements; blue boxes, *E. histolytica *genes; red boxes, *E. dispar *genes. GenBank accession numbers for each scaffold are shown above lines. Orthologous gene pairs between *E. histolytica *and *E. dispar *are connected by gray areas. Percent identity plots between nucleotide sequences DS571146 and DS550750 (positions 6 Kb – 19 Kb) and nucleotide sequences DS571146 and DS550750 (positions 32 Kb – 51 Kb) are shown above and below scaffolds respectively. Numbers indicate scaffold coordinates in Kb. Vertical gray lines depict the locations where synteny disappears.

During our comparative sequence analysis between *E. histolytica *and *E. dispar *we identified many syntenic break points rich in repetitive elements (Fig. 9) where similarity disappears within TEs (gray vertical lines in Fig. 9) suggesting that recombination occurred within repetitive regions. Therefore, it is likely that the striking enrichment of TEs in *E. histolytica *had contributed to its divergence from *E. dispar*.

The combined approach using intra- and inter-species comparative sequence analysis and TransposonPSI searches based on position-specific scoring matrices specific for different families of repetitive elements led to the identification of several novel TEs that had not been detected in previous studies [2-5] and showed that *E. histolytica *and *E. dispar *share most of their TE repertoire with the exception of Eh_ERE2. Whether the acquisition of Eh_ERE2 and/or the remarkable amplification of TEs contributed to *E. histolytica *pathogenicity remains to be elucidated.

## Conclusion

The present study provides the first insight into the composition and genomic organization of transposable elements for the newly generated assemblies of *E. histolytica*, *E. dispar *and *E. invadens*. Our analysis shows that the *E. histolytica *genome has three times more transposable elements than previously reported (20% of the genome and mean repeat density of 4 TEs every 10 Kb), and twice as much as its closest relative, *E. dispar*. Transposable elements are not uniformly distributed but have a tendency of being aggregated forming clusters. Repeat-clusters are frequently found at syntenic break points between *E. histolytica *and *E. dispar*, vouching for the contribution of transposable elements to genome instability and speciation. The expansion of LINE and SINE retroelements in the common ancestor to *E. histolytica *and *E. dispar *gave origin to three different families of LINE and SINE elements. We also describe two novel transposable elements, ERE1 and ERE2. ERE1 was present in the common ancestor of the three Entamoebas but it was expanded in the linage that led to *E. histolytica *and *E. dispar*, while ERE2 was acquired by *E. histolytica *after its divergence from *E. dispar*. All together the results presented in this study contribute to a better understanding about the genome architecture of these organisms and will help to clarify the processes that might have contributed to genome variation and speciation in these parasites, particularly between pathogenic *E. histolytica *and non-pathogenic *E. dispar*.

## Abbreviations

TE: transposable element; TSD: target site duplication; TIR: terminal inverted repeat.

## Authors' contributions

HL performed repeat identification, phylogenetic analyses, and genomic organization studies and wrote the manuscript. EC is the principal investigator for the genome projects of *E. histolytica*, *E. dispar *and *E. invadens*, coordinated and participated in the interpretation and discussion of results and contributed to the writing of the paper. MT carried out repeat identification analyses using RepeatScout and participated in the discussion of results and writing of the paper. BH contributed to the analysis and interpretation of TransposonPSI results. JW and NH significantly contributed to the interpretation of results and to the manuscript discussion. All authors read and approved the final manuscript.
